# Agent-based *post-hoc* correction of agricultural yield forecasts

**DOI:** 10.3389/frai.2026.1911884

**Published:** 2026-09-17

**Authors:** Matthew Beddows, Aiden Durrant, Georgios Leontidis

**Affiliations:** 1 School of Natural and Computing Sciences, University of Aberdeen, Aberdeen, United Kingdom; 2 School of Computing Sciences, University of East Anglia, Norwich, United Kingdom; 3 Department of Physics and Technology, UiT The Arctic University of Norway, Tromsø, Norway

**Keywords:** agricultural yield prediction, LLM agents, Moirai2, time series forecasting, XGBoost

## Abstract

Accurate crop yield forecasting in commercial soft fruit production is constrained by the data available in typical commercial farm records, which lack the sensor networks, satellite imagery, and high-resolution meteorological inputs that most state-of-the-art approaches assume. Even when provided with recent historical observations, existing forecasting models exhibit systematic, season-specific errors that growers can readily identify but that the models have no mechanism to self-correct. This work proposes a structured LLM agent framework that performs *post-hoc* correction of existing model predictions without requiring additional data or retraining, encoding agricultural domain knowledge across a five-tool pipeline covering seasonal phase detection, bias learning, range validation, cross-plot similarity search, and correction synthesis. The framework operates as a model-agnostic layer on top of any base forecaster, with each correction traceable to an explicit reasoning step. Evaluated on a proprietary strawberry yield dataset and a public USDA corn harvest dataset, agent refinement of XGBoost reduced MAE by 22% and MASE by 54% on strawberry, with consistent improvements across Moirai2 (MAE −25%, MASE −22%) and Random Forest (MAE −31%, MASE −61%) baselines. Using Llama 3.1 8B as the agent produced the strongest LLM-based corrections across all configurations, the Deterministic baseline was competitive with or outperformed Llama 3.1 8B on XGBoost and Random Forest, suggesting that domain-tool correction alone recovers much of the available gain. LLaVA 13B showed inconsistent gains, highlighting sensitivity to the choice of refinement model.

## 1 Introduction

Accurate yield forecasting is essential for labor allocation, cold storage, and retail contract fulfillment for UK strawberry producers (Celis et al., 2024), yet most state-of-the-art approaches assume sensor networks, satellite imagery, or high-resolution meteorological records that commercial growers simply do not have access to. This access problem is compounded by a structural one: approximately 95% of UK strawberries are grown under polytunnels (Batke et al., 2025), rendering satellite and UAV-based collection impractical regardless of cost. In practice, a typical farm record consists of a few years of weekly yield figures, basic plot metadata, and externally sourced weather data. This creates a persistent gap between what the literature demonstrates and what can realistically be deployed.

One way to address this is to acquire additional data, but this can often be difficult for growers due to both monetary and time costs, and this would also rule out years of previous historical data they cannot retroactively improve. Our method takes a different approach, reasoning more effectively over the data that is already available.

The core problem this work addresses is that existing forecasting models even when provided with recent historical observations as an input, continue to exhibit systematic errors, that growers can easily distinguish as errors. This work proposes that explicitly encoding this reasoning into a structured agent layer produces consistent improvements over the base model, without requiring additional data or retraining.

Figure 1 shows two characteristic failure modes. In both cases a grower would immediately reject the forecast: strawberries do not yield before flowering, and fields do not recover after clearance. The model has no mechanism to enforce either constraint. The model sees only numbers, it has no representation of where the crop is in its growing cycle.

**Figure 1:**
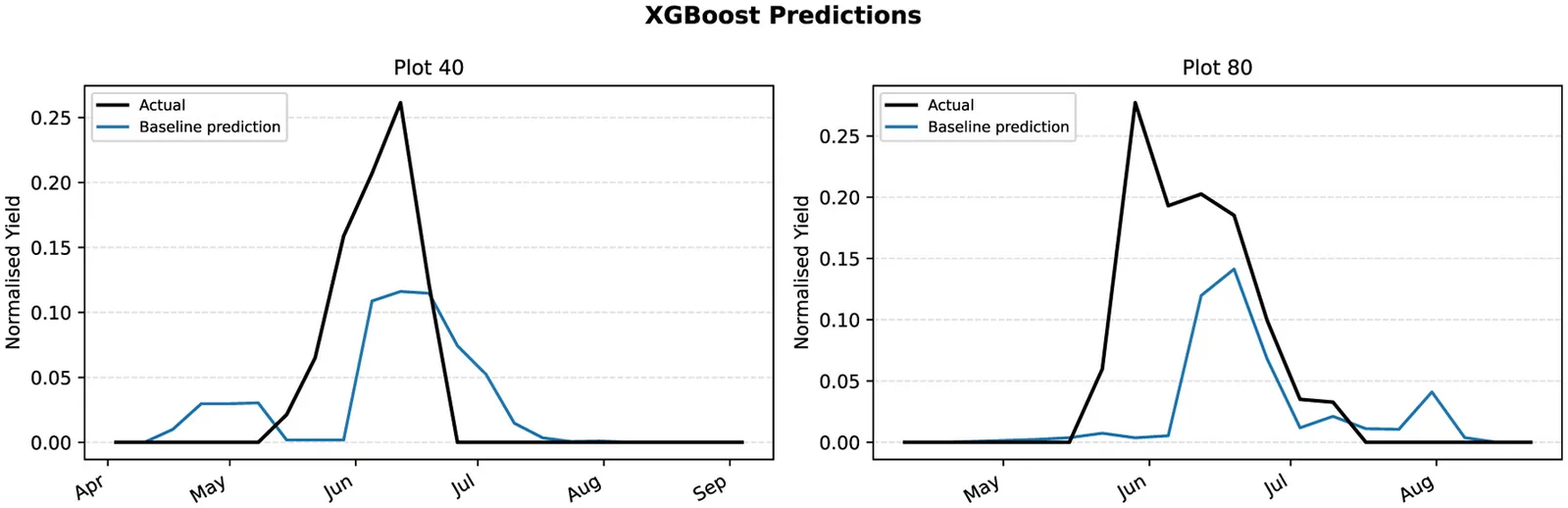
XGBoost research baseline predictions on two test plots, showing a pre-season spike **(left)** and a post-season spike **(right)**. Both illustrate how a model trained on windowed numerical features, without explicit seasonal constraints, can produce predictions that contradict the crop's growth cycle.

This is the gap the agent fills. The base model's prediction is taken as-is; the agent then checks it against the crop's current seasonal phase, recent error history, and the training distribution, and corrects it where the prediction contradicts known seasonal behavior. Figure 2 shows the overall system architecture.

**Figure 2:**
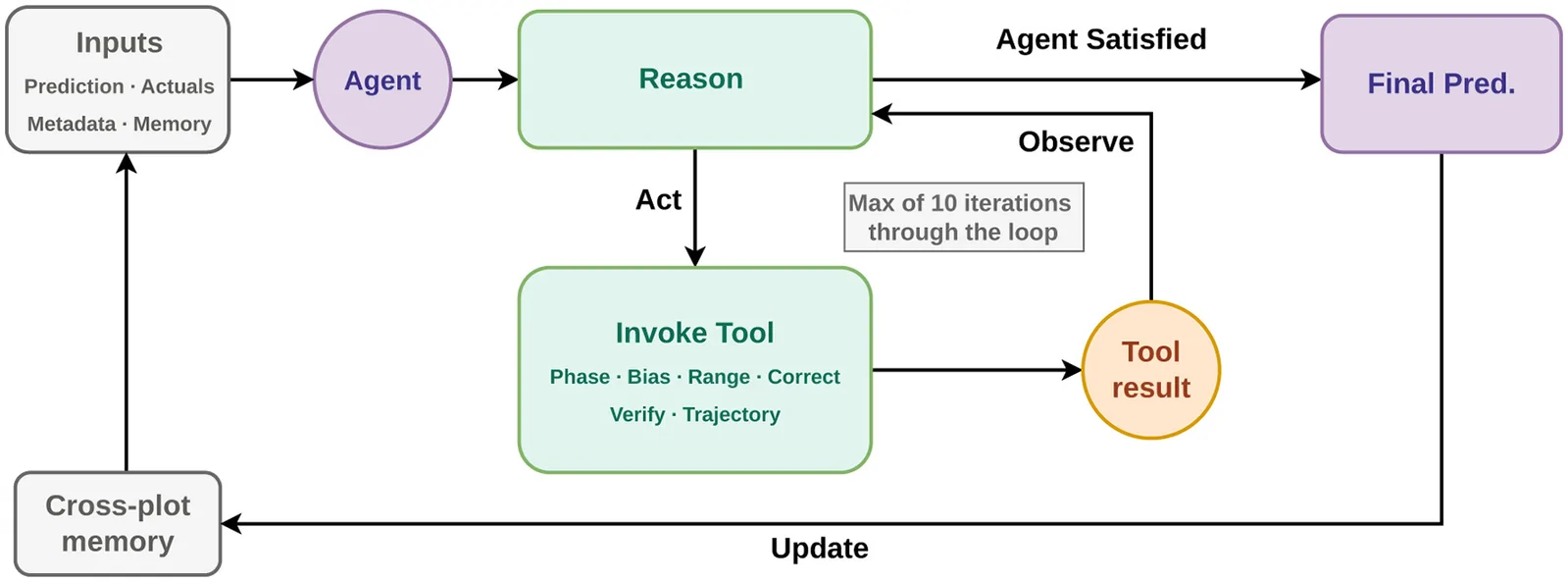
Overview of the agent pipeline. The ReAct loop iterates over the tool library to refine the raw prediction, the accepted correction is passed to the cross-plot memory, which is injected into the agent prompt on subsequent predictions.

### 1.1 Novelty and contributions

Our research demonstrates that an agent that provides LLM-based *post hoc* prediction correction can consistently improve yield forecasts in agricultural applications.

This work makes the following contributions:

- Proposes a structured LLM agent framework for *post-hoc prediction correction*, operating on top of an existing forecasting model rather than replacing it—a largely unexplored field in time series forecasting.
- The agent encodes agricultural domain knowledge into a fixed toolset, covering seasonal phase detection, bias learning, range validation, and correction synthesis, where each correction can be traced to a specific, inspectable reasoning step.
- The method is evaluated on two seasonal datasets of different crops from different origins. The datasets include a proprietary polytunnel strawberry dataset and the public USDA corn harvest dataset. The method achieves MAE reductions of up to 28% on strawberry and 24% on corn across three baseline forecasting models, demonstrating that the approach generalizes across domains and models.

## 2 Related work

### 2.1 Machine learning in agrifood

Globally, there has been substantial advancements in the integration of AI into agricultural practices, particularly in precision farming, crop yield forecasting, and logistics/resource management (Arévalo-Royo et al., 2025; Oliveira and Silva, 2023). However, even with all these developments, there are still many limitations that remain around data availability, system interoperability, model development, deployment, and adoption (Durrant et al., 2022; Chen et al., 2024; Cricelli et al., 2024).

Machine learning methods have already proven effective in many agricultural sectors (Alhnaity et al., 2019; Uppalapati et al., 2025; Suarez et al., 2024). In particular, some studies have highlighted the accuracy of these methods for predicting strawberry yields (Onoufriou et al., 2023; Liu S. et al., 2025).

Although these studies reported positive outcomes with transformer models and extensive datasets (Onoufriou et al., 2023; Lee et al., 2020), they depended on high-resolution information from irrigation infrastructure, meteorological stations, and high resolution harvest records. Often crop yield forecasting relies on extensive meteorological data, remote sensing information, and soil characteristics (Xiao et al., 2025), which are not available to many growers.

Often approaches have utilized satellite imagery and soil parameters (Chaudhary et al., 2021) or UAV-mounted cameras (Zheng et al., 2022). These methods have shown promise, but the UK Soft Fruit industry is very reliant on polytunnels, with 95% of UK strawberries being grown within them (Batke et al., 2025); this makes these solutions impractical. More modern techniques such as transformer-based time-series models have also been successful in crop yield forecasting. However, once again, these models rely on comprehensive and/or multi-modal datasets (Lin et al., 2023; Onoufriou et al., 2023). In our prior work, foundation time series models were combined with traditional ML techniques to navigate this constraint (Beddows et al., 2025); however this is limited to univariate data, which excludes a lot of important data growers routinely collect on their crops. This motivated our alternative approach: rather than requiring additional data modalities as a prerequisite, the system instead reasons more effectively over the inputs already available to the forecasting pipeline.

### 2.2 LLM agents for structured tasks

The rise of LLM agents has changed how complex tasks are automated by moving away from static prompts toward iterative loops of reasoning and tool use. This “ReAct” style approach Yao et al. (2022) allows a model to plan a step, execute an external query, and then adjust its logic based on the result. In time-series work, this has mostly involved using LLMs as sophisticated feature extractors or using fine-tuned models to interpret data directly (Jin et al., 2024). Models like Time-LLM (Jin et al., 2023), for instance, treat time-series data as a linguistic pattern-matching problem to generate forecasts. This trend extends into finance, where researchers are combining historical prices with news sentiment to build more explainable, multi-modal forecasting tools (Yu et al., 2023). Some have even used LLMs to “guess” the underlying structure of a dataset to help train Graph Neural Networks, often beating traditional deep learning benchmarks (Chen et al., 2023).

However, there is a skepticism regarding whether LLMs are actually “reasoning” or just executing complex pattern recognition. In many structured tasks, standard LLMs still struggle to beat small, specialized models that are purpose-built for relational logic (Li et al., 2024). Even if this “reasoning” debate is set aside, some models are still proving useful, for example, the DCATS framework (Yeh et al., 2025) uses an agent to clean and refine training data, which reportedly cuts error rates by 6% across various models. In the agricultural domain specifically, Mandiga et al. (2026) demonstrated that a ReAct-based agent with expert-designed tools substantially outperforms standalone LLMs on structured poultry nutrition queries, with tool-grounded responses reducing hallucination by 62% and improving numerical precision by over 160% compared to GPT-4o. Their work highlights that encoding domain knowledge directly into a fixed toolset, rather than relying on the LLM's parametric knowledge alone, is a meaningful design decision that determines whether an agent produces reliable outputs in specialist settings.

The current gap in the literature is that LLMs are almost always used before or during the forecasting process, for data prep or for the prediction itself. NutriCHAT (Mandiga et al., 2026) and similar systems ground the agent in domain knowledge at query time, but the prediction itself remains unchallenged. Very little work has been done on *post-hoc* correction, or using an agent to fix a prediction that has already been made. In many industries, the “base” forecasting models are already decent, but they often make systematic errors that they lack the context to fix. This research proposes an agent-based layer that sits on top of these existing predictors. Instead of asking the LLM to predict the yield, it is used it to apply seasonal and statistical logic to the model's output, using the same messy data growers rely on: yield history, plot metadata, and weather patterns.

## 3 Datasets

The method was evaluated on two seasonal datasets. A proprietary soft fruit yield dataset collected in partnership with a commercial grower Angus Soft Fruits, and a crop harvest dataset created from publicly available data from the USDA, chosen to demonstrate that the method is not dependent on domain-specific data infrastructure.

### 3.1 Angus soft fruits

To demonstrate real-world applicability, this work was collaborated on with Angus Soft Fruits Ltd, a large commercial supplier of soft fruit to UK and European retailers. The dataset comprises strawberry yield records from 2020 onward, covering 123 plots across 16 farms in Scotland and England. Variables include date, yield, farm and plot identifiers, plot acreage, variety, and tunnel type. Data from 2020 to 2022 form the training set and 2023 the test year, with 43 plots used for evaluation.

Yield records were supplemented with ERA5-Land reanalysis weather data matched to each farm's location and week, including temperature, dewpoint temperature, total precipitation, surface pressure, and wind components aggregated to a daily resolution. ERA5-Land was used in place of on-site weather observations, which were unavailable, and has been validated as a reliable substitute in similar agricultural contexts (Beddows and Leontidis, 2024).

### 3.2 USDA corn harvest

To validate the method on a second seasonal dataset which could be publicly accessed, weekly corn data was sourced from the USDA National Agricultural Statistics Service (NASS) QuickStats database across 38 US states from 2020 to 2023, with 2020–2022 used for training and 2023 held out as the test year. Three data items were retrieved, harvest progress (PCT HARVESTED), used as the primary yield signal, crop condition (PCT EXCELLENT and PCT GOOD), combined into a seasonal health indicator and planting progress (PCT PLANTED), used to characterize whether each state's season started early or late relative to historical norms. USDA reports harvest progress as a cumulative weekly percentage, consecutive values were differenced to recover a week-by-week harvest rate. The resulting series follows the same bell-curve structure as the strawberry data, rising from zero at the start of the season, peaking mid-autumn, and returning to zero once harvest is complete.

### 3.3 Feature engineering

A 2-week prediction horizon was applied, meaning the most recent yield observation available to the model at prediction time is from 2 weeks prior. For XGBoost and Random Forest, the input window covers weeks *t*−5 to *t*−2, creating a deliberate gap that reflects the real-world constraint of knowing only what has already been harvested. For the strawberry dataset ERA5 weather features were windowed identically. Categorical variables (tunnel type, farm and plot identifiers) were one-hot encoded. Rather than training on all available plots, the system selects the top *K* = 50 most similar historical plots by DTW distance as the training context for each test entity.

## 4 Methodology

The proposed system is a hybrid framework combining conventional ML forecasting with a Task-Oriented Autonomous Agent using a ReAct (Reason + Act) orchestration loop. Rather than building a more complex forecasting model, a reasoning layer was added on top of an existing one. A conventional ML model generates the initial prediction, and the agent then examines that prediction in context with recent yield history, the crop's current growth stage, systematic errors the model has been making, similar historical plots retrieved from a knowledge graph and data-driven guardrails derived from the training distribution to decide whether and how to correct it.

### 4.1 System pipeline overview

Figure 3 gives an overview of the full pipeline. The system operates in two phases: knowledge graph construction from historical training plots, followed by a week-by-week prediction loop in which the agent corrects each forecast in turn.

**Figure 3:**
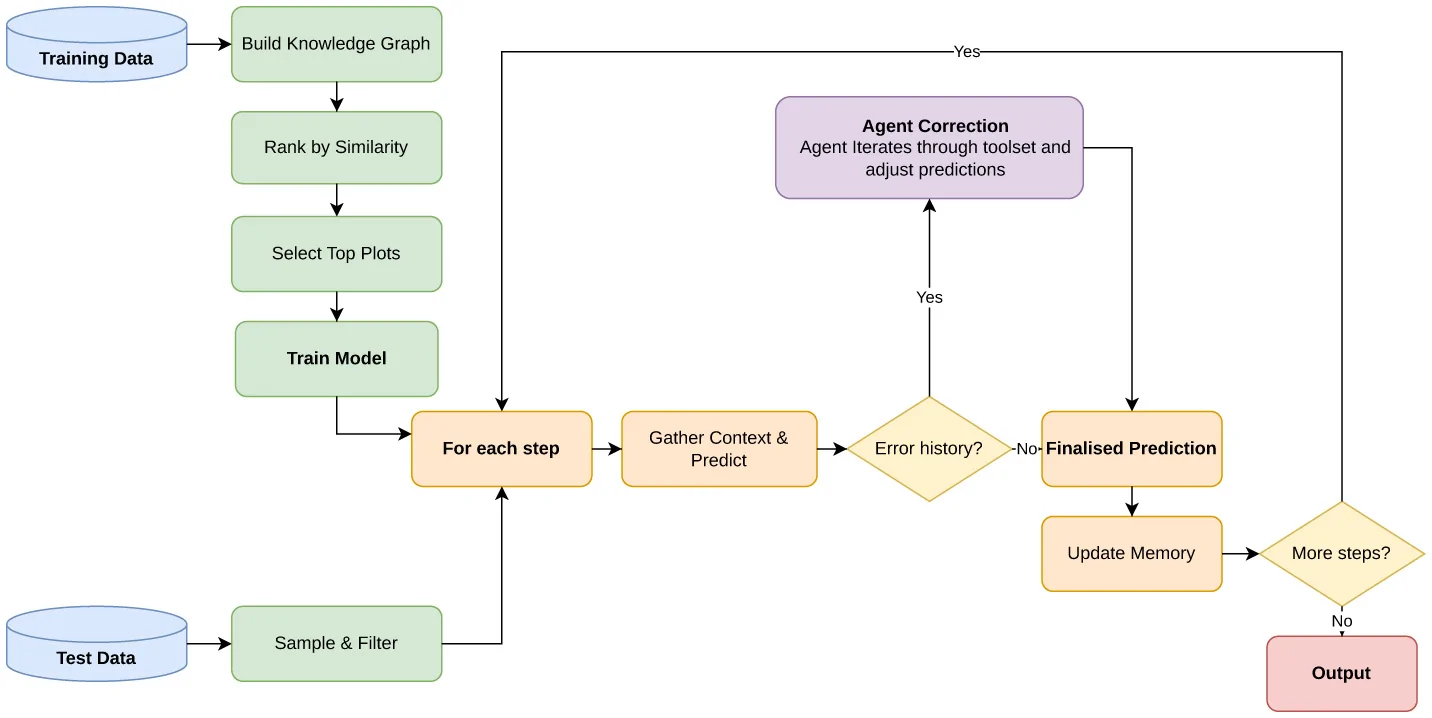
High-level pipeline overview. Training data is encoded into the knowledge graph; test data is prepared and fed into the weekly prediction loop one step at a time.

#### 4.1.1 Phase 1: knowledge graph construction

A grower's understanding of their operation is built up over many seasons, not reconstructed from scratch each year. The knowledge graph mirrors this by encoding the character of every training plot as a structured set of curve shape features, capturing not just raw yield figures but the geometric and seasonal properties that define how each plot behaves.

#### 4.1.2 Phase 2: prediction loop

As the season progresses, a grower's assessment of one plot informs how they read any other. The prediction loop replicates this by advancing all test plots 1 week at a time, allowing the agent to learn from plots already processed while never drawing on observations from future weeks. This lockstep structure ensures the agent's accumulated knowledge reflects only what would genuinely be available at each point in the season. The agent's reasoning loop, toolset, and correction pipeline are described in full in Section 4.5.

- **Observation:** at each correction step the agent receives the state vector *S* summarized in Table 1. The **Jump Distribution** is the empirical distribution of consecutive week-on-week ratios *y*_*t*_/*y*_*t*−1_ computed across all training plots, and summarized as *P*_01_–*P*_99_ percentiles is included as a data-driven guardrail for **Safety Verifier**.
- **Decision logic:** the agent selects tools based on the current state via a ReAct loop of up to 10 iterations. At each iteration it receives a prompt containing the current state and responds with a reason and a tool to call next. The loop exits early when the agent returns no further tools. The **Safety Verifier** is forced automatically after every correction call.

**Table 1:** Contents of the agent state vector *S* at each prediction step.

Component	Used by
Raw prediction and prediction interval	All tools
Recent actuals	Bias learning, phase detection, range validation
Recent predictions	Bias learning
Lookahead prediction	Phase detection
Forecast horizon	Phase detection
Seasonal position	Apply correction, position bias table
Known lagged error	Bias learning, position bias table
Entity metadata	Similarity search, agent prompt
Historical plot data	Range validation, safety verifier
Jump distribution	Safety verifier

### 4.2 Knowledge graph construction

A grower's accumulated knowledge of their plots is not stored as raw yield figures but as an intuitive sense of each crop's character: how volatile it is, when it typically peaks, how quickly it declines. The knowledge graph formalizes this by encoding each training entity as a node carrying two types of attributes: metadata from the data loader (farm, variety, plot size, etc.) and a set of curve shape features computed from the training time series, listed in Table 2.

**Table 2:** Curve shape features stored per KG node.

Feature	Formula	What it captures
Mean	ȳ	Average yield level
Std deviation	σ_*y*_	Spread of yield
CV	σ_*y*_/ȳ	Relative volatility
Volatility	σ(Δ*y*_*t*_)	Week-on-week smoothness
Peak position	i^*^/*N*	Where in season peak occurs (0–1)
Zero fraction	$1-\frac{1}{N}\sum 1\left[y_{t}>0\right]$	Proportion of off-season weeks
Early mean	ȳ_[0, *N*/3]_	Average yield in first third
Mid mean	ȳ_[*N*/3, 2*N*/3]_	Average yield in middle third
Late mean	ȳ_[2*N*/3, *N*]_	Average yield in final third
Growth pattern	see below	Seasonal shape classification
Harvest window	start, peak, end ISO week	Calendar timing of the season

The growth pattern is assigned by comparing third-by-third seasonal means: peak_middle if the mid mean exceeds both early and late by more than 10%; increasing if the late mean exceeds the early mean by more than 10%; decreasing if the late mean falls below 90% of the early mean; and flat otherwise.

At prediction time, these features serve three distinct purposes. *Similarity Search* queries the KG for training plots whose curve geometry most closely mirrors the current test entity, providing the agent with analogically grounded historical comparisons. *Dataset Profiling* reads zero_fraction and growth_pattern across all nodes to classify the dataset as seasonal or continuous, configuring the agent's phase logic accordingly. *Phase Detection* draws on the stored harvest window to provide the lookahead context required for false-start detection.

Test entities are kept out of the KG entirely to prevent data leakage. Their metadata is extracted separately and passed to the agent prompt only, never used for similarity retrieval.

### 4.3 Training plot selection

A grower carries season-to-season memory of how their plots typically behave, drawing on the years that most closely resemble the current one rather than treating all historical records as equally informative. The agent replicates this by ranking each training plot by its average dynamic time warping (DTW) similarity against all test plots, and retaining the top *K* = 50 most representative plots as the training context for the base model. As pairwise DTW comparisons are computationally expensive, this ranking is computed once and cached for reuse on subsequent runs.

### 4.4 Agent architecture and orchestration

A human expert does not correct a forecast in a single glance, they gather evidence, form a judgement, act on it, and then reassess. The agent mirrors this deliberative process through a ReAct loop of up to ten iterations per prediction week. Each iteration consists of a single LLM call followed by tool execution. The agent receives a structured prompt containing the current prediction, recent actuals, tool results accumulated so far, and any memory context carried forward from previous plots. It responds with a REASON block explaining its decision and a TOOLS block listing which tools to invoke next. The loop then parses this response, executes the requested tools, updates the agent state, and issues a fresh LLM call with the updated context. The loop exits early when the LLM returns no further tool calls, indicating the agent is satisfied with the correction. If ten iterations are reached without convergence, the last computed correction is accepted as the final output. In practice, most predictions resolve in two or three iterations: a diagnostic pass to detect the seasonal phase, learn positional bias, and validate the predicted range against historical bounds, followed by a correction pass to apply and verify the result. The 10-iteration ceiling exists as a safeguard against pathological cases rather than as an expected operating regime.

### 4.5 Prediction loop

#### 4.5.1 Dataset profiling

A human grower understands the nature of their crop and operation intuitively, but the agent is dataset-agnostic and must first characterize the data it is working with. Before any predictions are made, the agent inspects the knowledge graph to classify the dataset and configure itself accordingly. For each training entity, the fraction of zero-yield weeks is computed per year. Three entities are sampled and their per-year zero fractions and week-of-year profiles are passed to the LLM, which classifies the dataset as either zero_valley, where the series genuinely returns to zero during an extended off-season, or positive_floor, where yields remain above zero year-round. Where no LLM is available, a heuristic fallback applies: if the median per-year zero fraction across sampled entities exceeds 15%, the dataset is classified as zero_valley, and positive_floor otherwise.

#### 4.5.2 Week-by-week prediction loop

A grower does not assess all plots simultaneously before moving to the next week; they walk the crop round by round, building a picture of the season incrementally. The prediction loop mirrors this practice by advancing all test plots in lockstep, 1 week at a time, rather than completing a full plot before moving on. After each plot is processed, its partial curve is committed to the agent's plot history, so find_similar draws from a progressively richer retrieval pool as the run continues. The position bias table is similarly updated each week using the confirmed error from 2 weeks prior, once that lagged actual becomes available.

For each week, the system iterates over all test plots and generates a prediction for each before advancing. Each prediction uses the four most recently observed yields ending 2 weeks before the target date as its context window. A second prediction 1 week further ahead is generated in parallel, providing the agent with a view of the model's immediate expectation and enabling suppression of isolated noise spikes to zero where appropriate (see false_start, Section 4.6.2.2). Agent correction is withheld until at least one lagged actual has been recorded; correcting against an empty bias table would be uninformative and risks introducing noise before any systematic error pattern has emerged.

#### 4.5.3 Agent prompting

At each reasoning step, the agent receives a structured prompt containing the raw prediction, available tools with a record of which have already been called, accumulated position bias figures, and a compact set of ordering rules. Replies are constrained to a one-sentence justification and a single tool call, keeping each decision auditable and unambiguous. Any meta-strategy directives synthesized from prior plots are appended at the foot of the prompt (see Section 4.7).

### 4.6 The agent's tool library

The agent orchestrates a suite of expert-informed tools that function not as isolated scripts, but as a cohesive, sequential diagnostics pipeline. These tools represent holistic heuristics of the agricultural practice, providing the agent with mechanisms to diagnose, bound, and correct predictions statistically.

#### 4.6.1 Retrieval and contextualization: similarity search

Human experts naturally evaluate current crop performance by recalling historically similar seasons, whereas standard ML inference lacks this analogical reasoning. To replicate this, the *Similarity Search* retrieves three historical plots that mathematically mirror the current curve geometry of the target plot. Each plot is encoded as a feature vector, **V**, encompassing the relative volatility (CV), peak/trough positions (*t*^peak^, *t*^trough^), number of peaks ($\frac{\min\left(n^{\text{peaks}},5\right)}{5}$ capped at 5), and relative “spikiness” to the seasonal mean (*y*^peak^/ȳ), as given in Equation 1.


$$\begin{array}{l} v=\left[\text{CV},\;t^{\text{peak}},\;t^{\text{trough}},\;\frac{\min\left(n^{\text{peaks}},5\right)}{5},\;\frac{y^{\text{peak}}}{ȳ},\;\frac{ȳ}{y^{\text{peak}}}\right] \end{array}$$ (1)

By minimizing the Euclidean distance across these vectors *d*(*i, j*) = ||**v**_*i*_−**v**_*j*_||_2_, the agent is able to retrieve plots that share an underlying seasonal distribution, to identify proven historical yield trajectories for further analysis, rather than solely relying on localized raw yields.

#### 4.6.2 Diagnostic evaluation: bias learning and phase detection

These tools assess the baseline prediction in parallel to detect both statistical drift and seasonal phase violations.

##### 4.6.2.1 Bias learning

Statistical forecasts frequently drift into system-wide over or under estimates as the season progresses. In practice, the grower will notice the directional biases and apply a discount or premium to the models future outputs to account for this drift. The *Bias Learning* tool formalizes this intuition by evaluating recent predictions against observable lagged actual values. To prevent aggressive corrections based on localized noise, a directional bias is only applied if the ratio of mean error to mean absolute error exhibits a consistent trend (μ_*e*_>0.3 × μ_|*e*|_).

Once confirmed, the magnitude is estimated using the median ratio of actuals to predictions, increasing the robustness to outliers (Equation 2).


$$\begin{array}{l} \gamma =\text{median}\left(\frac{y_{\text{actual}}}{y_{\text{predicted}}}\right)\;\text{for }y>0. \end{array}$$ (2)

To prevent hallucinated positive yields post-harvest, this correction factor is exponentially decayed toward neutral as trailing zero-yield weeks accumulate, where *k* is the number of consecutive trailing zero weeks accumulate (Equation 3).


$$\begin{array}{l} \gamma^{\prime }=1+\left(\gamma -1\right)\cdot 0.5^{k}\;\text{where}k>0,\;\gamma >1 \end{array}$$ (3)

##### 4.6.2.2 Phase detection

An experienced grower would immediately identify a baseline model prediction as implausible if it contradicts the current phase of the season; a positive yield prediction before onset, or a rising forecast during a confirmed decline, is immediately suspect. Phase Detection formalizes this intuition by segmenting the yield curve into five operational phases derived from curve geometry: pre_season and ended at the zero-yield boundaries, ramping and declining on the rising and falling slopes, and peak near the seasonal maximum. The tool receives the four most recently observed yields, the raw prediction ŷ, the forecast horizon *h*, and a one-step-ahead prediction from a second model run. From this, it derives a phase-conditioned estimate ŷ_phase_ and a confidence score *c*∈[0, 1], both forwarded to Apply Correction where *c* controls how strongly ŷ_phase_ overrides the raw prediction.

##### 4.6.2.3 Seasonal datasets

Phases are evaluated in priority order and the first matching condition is returned. pre_season, where no non-zero yield exists, sets ŷ_phase_ = 0 with *c* = 0.90; a false_start sub-case is detected when the model predicts a positive yield but the one-step-ahead prediction is near-zero, indicating an isolated noise spike before onset (*c* = 0.85). ended, where one or more trailing zero weeks follow the last non-zero week *y*_*l*_, also sets ŷ_phase_ = 0, with confidence scaling as *c* = min(0.95, 0.75+0.05*k*) where *k* is the number of trailing zero weeks. The early phase, where only a single non-zero week has been observed, sets ŷ_phase_ = max(ŷ, *y*_*l*_) with low confidence (*c* = 0.35) to avoid undercutting the sole observation. During declining, a per-week decay rate (Equation 4) is fitted to post-peak observations and projected forward by horizon *h* (Equation 5):


$$\begin{array}{lll} \delta & = & {\left(\frac{y_{l}}{y_{\text{peak}}}\right)}^{1/\max\left(\Delta t,1\right)} \end{array}$$ (4)

$$\begin{array}{lll} {ŷ}_{\text{phase}} & = & y_{l}\cdot \delta^{h} \end{array}$$ (5)

with values below 1.0 floored to zero, and confidence growing with the active season window *n*_*a*_ (the span from first to last non-zero week, including any gaps) as given in Equation 6:


$$\begin{array}{l} c=\min\left(0.85,\;0.4+0.1\cdot n_{a}\right) \end{array}$$ (6)

Active growth phases (ramping, peak) defer to the model with ŷ_phase_ = ŷ, as the yield trajectory remains least predictable, with peak carrying a moderate confidence of *c* = 0.40 and ramping fully trusting the model at *c* = 0.0.

##### 4.6.2.4 Continuous datasets

On continuous datasets, where no seasonal bell curve exists, the tool instead splits the recent window in half and compares the means of each half:


$$\begin{array}{l} \text{change}=\frac{{ȳ}_{\text{second}}-{ȳ}_{\text{first}}}{\left|{ȳ}_{\text{first}}\right|} \end{array}$$ (7)

A change exceeding +10% is classified as trending_up (Equation 7), below −10% as trending_down, and otherwise stable. In all three cases ŷ_phase_ = ŷ. Confidence is capped at 0.75 and scales with the magnitude of the change, so a weak trend carries little weight.

#### 4.6.3 Empirical bounding: range validation

Domain experts possess an intuitive understanding of plausible weekly growth rates, and will immediately reject a forecast that suggests an unprecedented leap that defies the known crop physiology. Therefore, *Range Validation* acts as a pre-correction filter, computing the fractional change from the last observed yield to the new prediction. The change is evaluated against the 10th and 90th percentiles of the historical changes observed at the exact same seasonal position (Equation 8).


$$\begin{array}{l} \text{Δ}\%=\frac{ŷ-y_{t-1}}{y_{t-1}}\in \left[P_{10}-0.5,\;P_{90}+0.5\right] \end{array}$$ (8)

Predictions breaching these empirical boundaries are clamped to the nearest plausible value, albeit with a 50% buffer outside the historical range. If a predicted change exceeds these limits, it is mathematically clamped to ŷ = *y*_*t*−1_·(1+*P*_90_) for an upward breach, or ŷ = *y*_*t*−1_·(1+*P*_10_) for a downward breach. However, if fewer than three historical comparisons are available at the current position, the tool defers and returns in-range by default. This ensures the proposed change is historically plausible before any further synthesis occurs.

#### 4.6.4 Synthesis: apply correction

An expert must weigh conflicting pieces of evidence, prioritizing physical constraints over statistical patterns. This tool integrates the empirical bounds and diagnostic outputs described above to resolve the final corrected value (ŷ^*^) through a strict three-tiered priority cascade, stopping as soon as a condition is met to prevent cascading errors. At the highest priority is the *Physical Limit Check*: if an implausible leap is flagged, the value is clamped to the nearest historical boundary, superseding all other logic. If the physical limits are respected, the system moves to the *Phase Check*. Here, if phase detection confidence is high (*c*≥0.5), the raw prediction is blended with the phase-conditioned estimate using the formula ${ŷ}^{*}=c\cdot {ŷ}_{\text{phase}}+\left(1-c\right)\cdot {ŷ}_{\text{raw}}$. When this phase blend is triggered, statistical multipliers are intentionally suppressed to avoid double-correction. Finally, if neither physical nor phase constraints are triggered, the system defaults to *Statistical Fine-Tuning*, applying the position bias correction and learned bias multiplier to adjust the final output.

#### 4.6.5 Verification: safety verifier and trajectory evaluation

Even human expert interventions require sanity check to ensure adjustments do not result in catastrophic algorithmic drift. The *Safety Verifier* prevents agent hallucinations by analyzing the final correction ratio ($R={ŷ}^{*}/{ŷ}_{\text{raw}}$) alongside absolute limits. Results that fall in the warning zone–where *R*∈[*P*_01_, *P*_05_] or [*P*_95_, *P*_99_] are flagged in the output logs but not overridden, allowing unusual but historically possible corrections to pass through. However, if the ratio exceeds the extreme 1st or 99th percentiles of the training distribution, predicts positive yields during dormant off-seasons, or exceeds three times the historical maximum the verifier forcibly overrides the agent and clamps the output to predefined safe boundaries. This boundary is defined as the *historical maximum clamp* where if ŷ^*^>3 × hist_max (where hist_max is the highest value seen across all training plots), the corrected value is immediately clamped to hist_max.

Simultaneously, *Trajectory Evaluation* acts as a self-critique mechanism to ensure the corrected value aligns with recent trends and the expected seasonal phase. It projects a plausible range for the upcoming week based on the last three observed yields, calculating a 30% tolerance above and below the trend-projected value: [ŷ_lo_, ŷ_hi_] =[0.7·min(*y*_*t*_, *y*_*t*_(1+δ)), 1.3·max(*y*_*t*_, *y*_*t*_(1+δ))], where the fractional trend slope $\delta =\left(y_{t}-y_{t-2}\right)/\left(2\cdot \bar{w}\right)$ is normalized by the 3-week mean $\bar{w}$.

The tool evaluates the correction against this range to issue a verdict, returning “consistent” if the value is in-range and phase-sound; “trend contradiction” if it aligns with the current phase but exceeds the mathematical range; and “phase contradiction” if it opposes the current seasonal phase, such as a downward correction during a confirmed ramping phase. If a contradiction in either trend or phase is detected, it triggers an *Iterative Adjustment*. This prompts the agent to dynamically recalibrate by choosing a revised blend weight (*w*′), allowing it to soften an overcorrection or adjust an undercorrection based on the specific failure mode. The agent then re-executes the correction pipeline using the updated formula: ${ŷ}^{*}\leftarrow w^{\prime }\cdot {ŷ}_{\text{phase}}+\left(1-w^{\prime }\right)\cdot {ŷ}_{\text{raw}}$. To ensure stability and prevent runaway loops, this iterative adjustment is strictly capped at a maximum of two attempts per prediction week.

### 4.7 Cross-plot persistent memory

Up to this point, the correction pipeline has functioned as a reactive system: detecting phases, enforcing physical limits, and smoothing trajectories based purely on the immediate context of a single plot. However, if the agent resets its context after every plot, it remains blind to broader systematic errors and risks repeating the same correction mistakes across the dataset. To solve this, the pipeline is augmented with a long-term memory mechanism.

First, a *Position Bias Table* continuously records percentage prediction errors mapped to specific spatial-temporal coordinates (week-of-year and season-progress). This allows the system to learn and proactively correct when the base model systematically fails at specific calendar milestones. Second, the *Plot History* archives the geometric shapes and final correction outcomes of every completed plot, continually expanding the dynamic retrieval corpus for the Similarity Search tool.

Finally, a *Meta-Strategy Reflection* protocol occurs at regular intervals. The agent processes a summary of recent correction outcomes, assessing tool efficacy and impact on mean absolute error. It synthesizes these findings into abstract strategic directives such as noting the counter-productivity of a specific tool during early growth, which are permanently injected into subsequent prompts. This enables the agent to autonomously refine its experiential heuristics over the lifespan of the dataset run.

### 4.8 Experimental setup

Three LLMs were evaluated for agent reasoning: llama3.1:8b, qwen2.5:7b, are text-based instruction-following models suited to structured numerical reasoning tasks. Llava:13b is a vision-language model included to examine the sensitivity of the framework to the choice of LLM model for refinement. Its pretraining is oriented toward image understanding rather than structured numerical reasoning, which makes it mismatched to this task. Where LLaVA produces inconsistent corrections and often degrades performance, this highlights that the choice of LLM model for refinement is a meaningful design decision. The agent framework provides the structure, but the underlying LLM must be capable of reliably following instructions with numerical context to be effective. All three models were hosted locally via Ollama with no external API calls. Baselines are XGBoost, Random Forest, and Moirai2 without agent correction.

All models were queried using Ollama's default sampling parameters (temperature 0.8, top_p 0.9) with no custom configuration applied. The seeds (21, 42, 256) control forecasting model initialization and data splitting; LLM sampling variability is captured implicitly across the three runs.

#### 4.8.1 Deterministic baseline

To isolate the contribution of the LLM from the contribution of the tool pipeline itself, a no-LLM deterministic condition is included. This configuration executes the same five tools in a fixed sequential order: learn_bias computes a multiplicative bias correction from recent prediction errors; detect_phase returns a phase-conditioned estimate and confidence score; validate_range clamps the prediction to empirically plausible bounds; apply_correction blends the phase estimate and bias multiplier using the confidence score directly; and verify_correction applies the safety clamp. No LLM is consulted at any step. It serves as a direct test of whether the domain knowledge encoded in the tools is sufficient on its own, independent of the language model.

### 4.9 Forecasting models

Three base forecasting models are evaluated as the prediction backbone, spanning classic gradient-boosted approaches and a modern zero-shot foundation model.

**XGBoost** is a gradient-boosted tree model trained on windowed yield and weather features. Despite lacking inherent temporal structure, it has proven competitive in yield forecasting tasks (Beddows and Leontidis, 2024) and outperforms specialized time series models such as ARIMA in similar settings (Alim et al., 2020). Three separate models are trained, one per quantile (*q*_10_, *q*_50_, *q*_90_), using quantile loss to produce a lower bound, median point forecast, and upper bound for each prediction. This gives the agent a direct signal of model uncertainty, and provides actionable prediction intervals for operational planning, since a grower needs to know the range of plausible outcomes rather than a single figure.

**Random Forest** is an ensemble method that aggregates predictions across multiple decision trees, and has been widely applied to crop yield forecasting (Prasad et al., 2021). It is trained on the same windowed yield and weather features as XGBoost, producing a single point forecast per prediction step.

**Moirai 2** (Liu C. et al., 2025) is a zero-shot time series foundation model pre-trained on 36 million series comprising approximately 295 billion observations. Unlike its predecessor (Woo et al., 2024), it adopts a decoder-only autoregressive architecture with quantile-based predictions and multi-token prediction, improving both probabilistic accuracy and inference efficiency.

## 5 Results

### 5.1 Overall performance

Table 3 reports normalized MAE, RMSE, and MASE across all evaluated configurations. Values represent the means across three random seeds, with standard deviations shown below each entry. The Moirai2 baseline uses a fixed pre-trained checkpoint, resulting in negligible seed variation for strawberry (±0.000) and minimal variation for corn. MASE is scaled by the two-step naive forecast ŷ*t* = *yt*−2, matching the 2-week prediction horizon used throughout; values below 1.0 indicate the model outperforms the naive baseline on that metric.

**Table 3:** Performance comparison across three random seeds (21, 42, 256).

		Angus strawberry			USDA corn		
Baseline	Refinement	MAE	RMSE	MASE	MAE	RMSE	MASE
**XGBoost**	None	0.0134	0.0432	2.834	0.0243	0.0719	0.674
		±0.0004	±0.0006	±0.263	±0.0001	±0.0002	±0.003
	Llama 3.1 8B	**0.0105**	**0.0392**	1.310	0.0239	0.0734	0.662
		±0.0001	±0.0002	±0.132	±0.0003	±0.0013	±0.009
	LLaVA 13B	0.0135	0.0466	3.236	0.0249	0.0753	0.686
		±0.0013	±0.0075	±0.291	±0.0008	±0.0042	±0.023
	Qwen 2.5 7B	0.0114	0.0405	2.204	0.0241	0.0723	0.666
		±0.0004	±0.0005	±0.539	±0.0003	±0.0015	±0.007
	Deterministic	0.0111	0.0404	**1.275**	**0.0237**	**0.0717**	**0.652**
		±0.0001	±0.0001	±0.049	±0.0000	±0.0002	±0.000
**Moirai2**	None	0.0161	0.0519	1.099	0.0496	0.1178	1.404
		±0.0000	±0.0000	±0.000	±0.0003	±0.0003	±0.000
	Llama 3.1 8B	**0.0121**	**0.0442**	**0.861**	**0.0379**	**0.0991**	**1.073**
		±0.0001	±0.0003	±0.014	±0.0000	±0.0001	±0.002
	LLaVA 13B	0.0163	0.0564	1.114	0.0505	0.1255	1.439
		±0.0015	±0.0099	±0.098	±0.0040	±0.0155	±0.127
	Qwen 2.5 7B	0.0138	0.0477	0.970	0.0425	0.1063	1.205
		±0.0001	±0.0005	±0.011	±0.0002	±0.0004	±0.005
	Deterministic	0.0123	0.0442	0.906	0.0399	0.1022	1.134
		±0.0000	±0.0000	±0.000	±0.0000	±0.0000	±0.000
**Random Forest**	None	0.0159	0.0433	4.310	0.0266	**0.0686**	0.734
		±0.0005	±0.0020	±0.040	±0.0001	±0.0035	±0.002
	Llama 3.1 8B	**0.0109**	**0.0389**	1.670	**0.0237**	0.0710	0.683
		±0.0001	±0.0003	±0.251	±0.0001	±0.0004	±0.043
	LLaVA 13B	0.0147	0.0420	4.841	0.0273	0.0774	0.753
		±0.0000	±0.0001	±0.128	±0.0016	±0.0106	±0.044
	Qwen 2.5 7B	0.0130	0.0397	4.284	0.0257	0.0708	0.714
		±0.0003	±0.0003	±0.199	±0.0002	±0.0009	±0.004
	Deterministic	0.0113	0.0400	**1.263**	0.0239	0.0712	**0.655**
		±0.0001	±0.0001	±0.006	±0.0000	±0.0001	±0.001

Mean values align with model name; ±SD shown below. All metrics normalized. Bold values denote highest performing model, i.e. lowest error, within each baseline group.

On strawberry, configurations with XGBoost and Random Forest baselines remain above 1.0. However, with long periods of zero yield at the start and end of the season, the naive forecast naturally achieves a low error score, predicting zero when the crop is not growing, which raises the bar for any corrected forecast to beat it.

### 5.2 Effect of agent refinement on XGBoost

Llama 3.1 8B, Qwen 2.5 7B, and the Deterministic baseline improved on the unrefined XGBoost baseline across both datasets; LLaVA 13B did not. On the Angus Strawberry dataset, Llama 3.1 8B achieved the largest MAE reduction, reducing MAE from 0.0134 to 0.0105 (−21.6%) and MASE from 2.834 to 1.310 (−53.8%). The Deterministic baseline achieved a comparable MASE reduction (2.834 → 1.275, −55.0%) with slightly higher MAE (0.0111). Qwen 2.5 7B followed, reducing MAE to 0.0114 and MASE to 2.204. LLaVA 13B degraded performance on strawberry, increasing both MAE (0.0134 → 0.0135) and MASE (2.834 → 3.236).

On USDA Corn, gains were smaller, consistent with the lower baseline error. The Deterministic baseline achieved the best results across all three metrics (MAE 0.0237, RMSE 0.0717, MASE 0.652). Llama 3.1 8B achieved MAE 0.0239 and MASE 0.662. Qwen 2.5 7B produced a modest MAE reduction to 0.0241 (MASE 0.666). LLaVA 13B degraded all three metrics on corn (MAE +2.5%, RMSE +4.7%, MASE 0.674 → 0.686), consistent with its vision-language pretraining providing limited benefit for structured numerical correction.

### 5.3 Effect of agent refinement on Moirai2

The Moirai2 foundation model provided a stronger strawberry baseline than XGBoost in MASE terms (1.099 vs. 2.834), but a weaker corn baseline (MAE 0.0496 vs. 0.0243). Llama 3.1 8B, Qwen 2.5 7B, and the Deterministic baseline improved results on both datasets; LLaVA 13B did not.

On strawberry, Llama 3.1 8B reduced MAE by 24.8% (0.0161 → 0.0121) and MASE by 21.7% (1.099 → 0.861). The Deterministic baseline achieved a similar MAE reduction (0.0161 → 0.0123, −23.6%) and MASE of 0.906. Qwen 2.5 7B achieved a 14.3% MAE reduction and brought MASE to 0.970. LLaVA 13B marginally degraded both metrics (MAE 0.0161 → 0.0163, MASE 1.099 → 1.114).

On corn, the weaker Moirai2 baseline gave the agent more to correct. Llama 3.1 8B reduced MAE by 23.6% (0.0496 → 0.0379) and MASE by 23.6% (1.404 → 1.073). The Deterministic baseline achieved a 19.6% MAE reduction (MASE 1.134). Qwen 2.5 7B achieved a 14.3% MAE reduction (MASE 1.205). LLaVA 13B again degraded performance (MAE +1.8%, MASE 1.404 → 1.439). Figure 4 illustrates the correction pattern for Random Forest + Llama 3.1 8B across both datasets.

**Figure 4:**
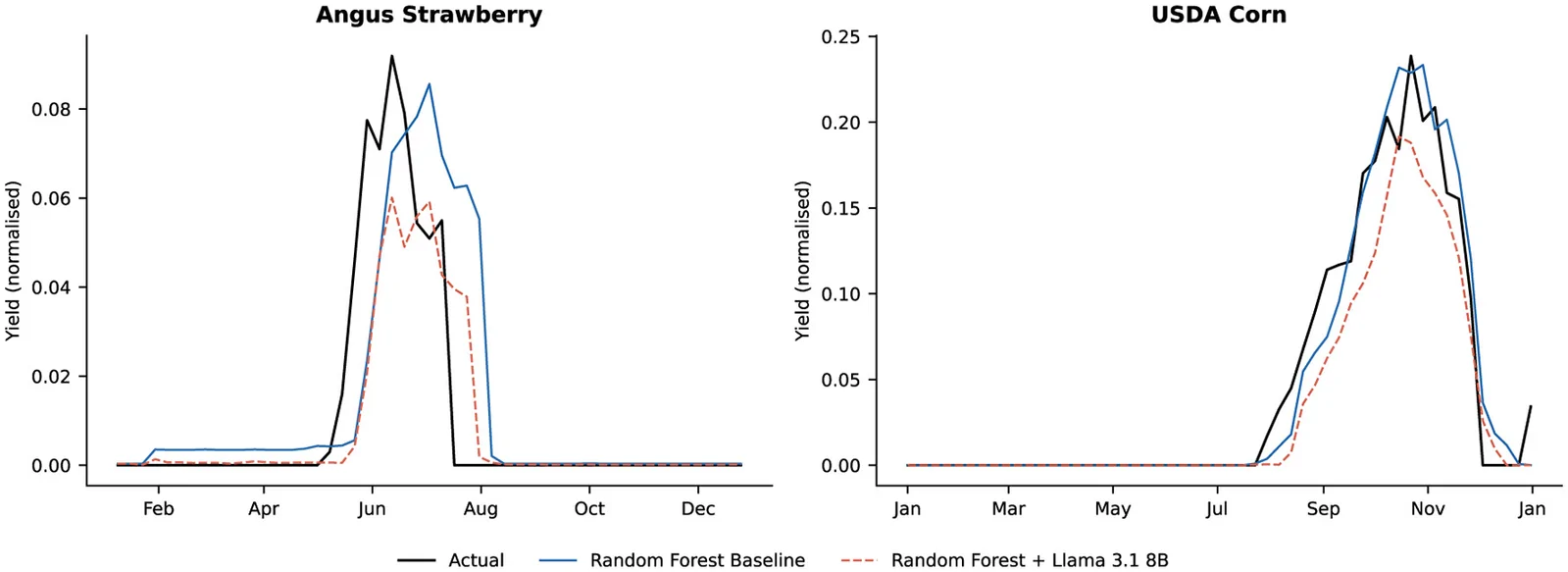
Random Forest and Random Forest + Llama 3.1 plotted against the actual yield (normalized), on the Angus strawberry and USDA corn datasets.

### 5.4 Effect of agent refinement on Random Forest

The Random Forest baseline has a high MASE on strawberry (4.310) relative to XGBoost and Moirai2, reflecting RF's tendency to predict small non-zero yields during the off-season. This provides the agent with a clear correction signal. The Deterministic baseline achieved the strongest MASE reduction of any configuration in the study: MASE −70.7% (4.310 → 1.263, MAE 0.0113). Llama 3.1 8B also produced a large reduction: MAE −31.4% (0.0159 → 0.0109) and MASE −61.3% (4.310 → 1.670). Qwen 2.5 7B achieved an 18.2% MAE reduction (MASE 4.284), while LLaVA 13B improved MAE (−7.5%) but marginally worsened MASE (4.310 → 4.841).

On the corn dataset, the RF baseline performed better (MAE 0.0266, MASE 0.734). The Deterministic baseline achieved the best MASE (0.655), with Llama 3.1 8B close behind (MAE −10.9%, MASE 0.683). Qwen 2.5 7B produced modest gains (−3.4% MAE, MASE 0.714) and LLaVA 13B degraded performance on corn (+2.6% MAE, +12.8% RMSE), consistent with its pattern of unreliable corrections on the structured corn signal. Figure 4 illustrates the correction pattern for Random Forest + Llama 3.1 8B across both datasets.

### 5.5 Cross-model analysis

Llama 3.1 8B was the most effective LLM-based refinement model across all configurations, achieving the best MAE and MASE among the three LLMs in every baseline/dataset combination. The Deterministic baseline was competitive with or outperformed Llama 3.1 8B on XGBoost and Random Forest configurations, where the systematic nature of the errors makes fixed correction rules highly effective. Llama 3.1 8B held a clear advantage on Moirai2, where the base model's errors are less predictable and benefit more from the LLM's flexible reasoning. Qwen 2.5 7B consistently scored second among the LLMs, producing moderate improvements. LLaVA 13B was the weakest and most inconsistent model, degrading results across both datasets for XGBoost and Random Forest, and on corn for Moirai2. Figure 5 shows the per-plot distribution of corrections for XGBoost + Llama 3.1 8B, with the majority of plots falling below the diagonal on both datasets.

**Figure 5:**
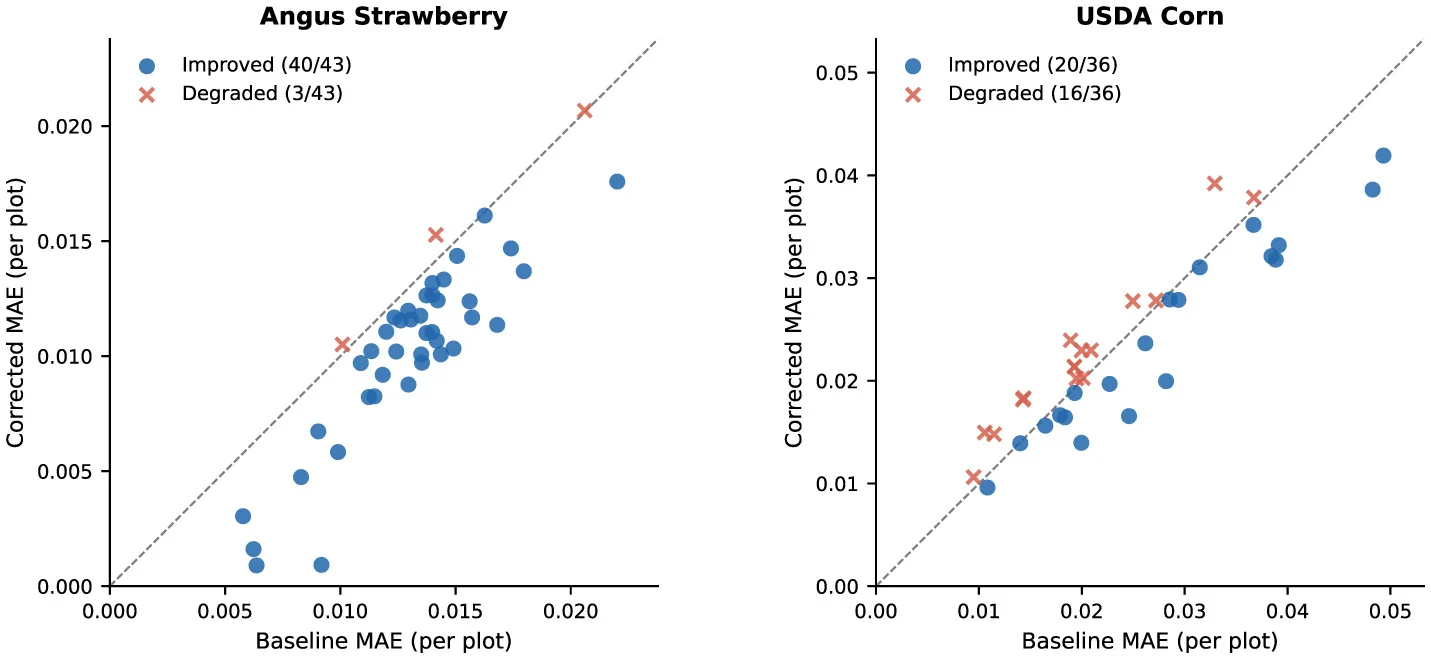
Per-plot MAE before and after agent correction (XGBoost + Llama 3.1 8B). Points below the diagonal indicate improvement. Blue circles = improved plots; red crosses = degraded plots.

### 5.6 Agent tool utilization

On average, the agent invokes 6.4 tools per prediction. Table 4 breaks this down by tool and phase. The analysis phase accounts for the bulk of calls: learn_bias and detect_phase are invoked on virtually every prediction (99.6%), providing the bias estimate and growth-phase context that anchor the correction. find_similar (84.1%) and validate_range (73.0%) are called conditionally, the agent skips them when historical analogs are sparse or the proposed correction falls within expected bounds without further checks.

**Table 4:** Agent tool invocation frequency across the 233 predictions altered by the agent on the strawberry dataset (XGBoost + Llama 3.1 8B).

Phase	Tool	Call rate
Analysis	learn_bias	99.6%
	detect_phase	99.6%
	find_similar	84.1%
	validate_range	73.0%
Correction	apply_correction	98.7%
	adjust_correction	19.7%
Verification	evaluate_trajectory	67.8%
	verify_correction	98.7%

Correction application (apply_correction, 98.7%) is near-universal, followed by evaluate_trajectory (67.8%), which assesses whether the corrected value is consistent with the seasonal trend to date. When the trajectory assessment is unfavorable, the agent invokes adjust_correction to refine the correction (19.7% of all predictions, or roughly one in three trajectory evaluations). verify_correction closes the loop on 98.7% of predictions, confirming the final value is physically plausible before it is accepted.

### 5.7 Ablation study

To quantify the contribution of each agent tool, a leave-one-out ablation on the best-performing configuration (XGBoost baseline, Llama 3.1 8B refinement) across both datasets was conducted. In each combination a single tool is disabled while the remainder of the pipeline operates normally. evaluate_trajectory and adjust_correction are disabled together, as the latter has no meaning without the former. Table 5 reports the corrected MAE, RMSE, and MASE for each condition. Table 6 reports the reverse ablation, where each tool is enabled in isolation, confirming that detect_phase alone accounts for the majority of the pipeline's correction capability.

**Table 5:** Ablation study: effect of removing individual agent tools.

	Angus strawberry			USDA corn		
Configuration	MAE	RMSE	MASE	MAE	RMSE	MASE
Full system	0.0105	0.0399	1.3212	0.0235	0.0714	0.6593
−learn_bias	0.0101	0.0388	1.2220	0.0246	0.0746	0.6798
−detect_phase	0.0222	0.0895	3.8581	0.0356	0.1261	1.0029
−validate_range	0.0105	0.0392	1.2664	0.0240	0.0744	0.6643
−find_similar	0.0103	0.0390	1.1926	0.0236	0.0721	0.6518
−evaluate_trajectory	0.0100	0.0387	1.1899	0.0252	0.0750	0.7047
−verify_correction	0.0109	0.0389	1.8391	0.0242	0.0727	0.6703
None (XGBoost baseline)	0.0131	0.0433	3.2057	0.0244	0.0721	0.6705

XGBoost baseline, Llama 3.1 8B, leave-one-out per condition.

**Table 6:** Reverse ablation study: effect of enabling a single agent tool in isolation.

	Angus strawberry			USDA corn		
Configuration	MAE	RMSE	MASE	MAE	RMSE	MASE
Full system	0.0105	0.0399	1.3212	0.0235	0.0714	0.6593
learn_bias only	0.0231	0.0891	3.8561	0.0377	0.1335	1.0672
detect_phase only	0.0101	0.0389	1.5381	0.0275	0.0805	0.7758
validate_range only	0.0125	0.0413	3.1375	0.0240	0.0701	0.6573
find_similar only	0.0129	0.0421	3.1987	0.0244	0.0721	0.6693
evaluate_trajectory only	0.0129	0.0421	3.1950	0.0244	0.0721	0.6693
verify_correction only	0.0129	0.0421	3.1949	0.0241	0.0716	0.6727
None (XGBoost baseline)	0.0131	0.0433	3.2057	0.0244	0.0721	0.6705

XGBoost baseline, Llama 3.1 8B.

Removing detect_phase causes the largest degradation across both datasets, MAE increases by 72.0% on strawberry (0.0105 → 0.0222) and 45.7% on corn (0.0235 → 0.0356) compared to the full system, with RMSE more than doubling on strawberry (0.0399 → 0.0895). This confirms that phase detection is the load-bearing component of the pipeline: without it, the agent lacks the context needed to reason about whether a prediction error stems from a correctable trend or a local deviation, causing corrections to become unreliable.

Removing verify_correction produces a notable MASE increase on strawberry (1.3212 → 1.8391) with only minor MAE change (+3.8%). Notably, several ablations marginally outperform the full system on MAE, likely reflecting noise at this scale of improvement rather than genuine redundancy. Taken together, the ablation suggests the pipeline is robust to the removal of any single secondary tool, but critically dependent on detect_phase to anchor the correction process.

### 5.8 Computational cost analysis

Without agent correction, the base XGBoost model completes inference in 31.34 s on the strawberry dataset (43 plots, 2,193 predictions) and 24.42 s on corn (36 plots, 1,908 predictions). Enabling Llama 3.1 8B correction increases wall time to 2.83 h and 2.44 h respectively, reflecting the cost of the ReAct loop issuing over 20,000 LLM calls per dataset, averaging 827 prompt tokens and 40 output tokens per call.

All inference was performed locally on an NVIDIA GeForce RTX 3070 (8 GB). LLM prompts were sent over a local network tunnel to a separate machine with an NVIDIA GeForce RTX 3090 (24 GB), with the responses returned to the host machine for use in the correction pipeline.

## 6 Discussion

The magnitude of agents prediction improvement correlates with baseline error. Combinations where the baseline model has lower accuracy (e.g., RF on strawberry, Moirai2 on corn) show the largest gains in performance. This is consistent with the agent's role as a post prediction corrector, the tools for **bias learning** and **phase detection** are most informative when the baseline prediction diverges substantially from expected phase patterns. Where the baseline prediction is already accurate (XGBoost on corn, MASE ≈0.67), the agent's room for improvement is limited and the corrections it makes are expectedly smaller.

The choice of refinement model matters. Llama 3.1 8B produced the strongest corrections in every baseline/dataset combination. The deterministic rule-based agent performed comparably to Llama in several configurations, suggesting that structured correction rules alone recover much of the available gain. LLaVA 13B was the weakest and most inconsistent: it improved strawberry results across all baselines but degraded corn performance for XGBoost and Random Forest, indicating sensitivity to the underlying LLM's ability to follow structured numerical instructions. Future work could investigate which plot, crop or season characteristics predict when agent corrections are most beneficial.

Figure 6 shows the per-plot MAE improvement distribution for XGBoost + Llama 3.1 8B across both datasets. The majority of plots benefit from the agents corrections, but a minority are degraded. Instances of the model degrading the predictions is mostly on the corn dataset, where LLaVA 13B produced the largest individual losses.

**Figure 6:**
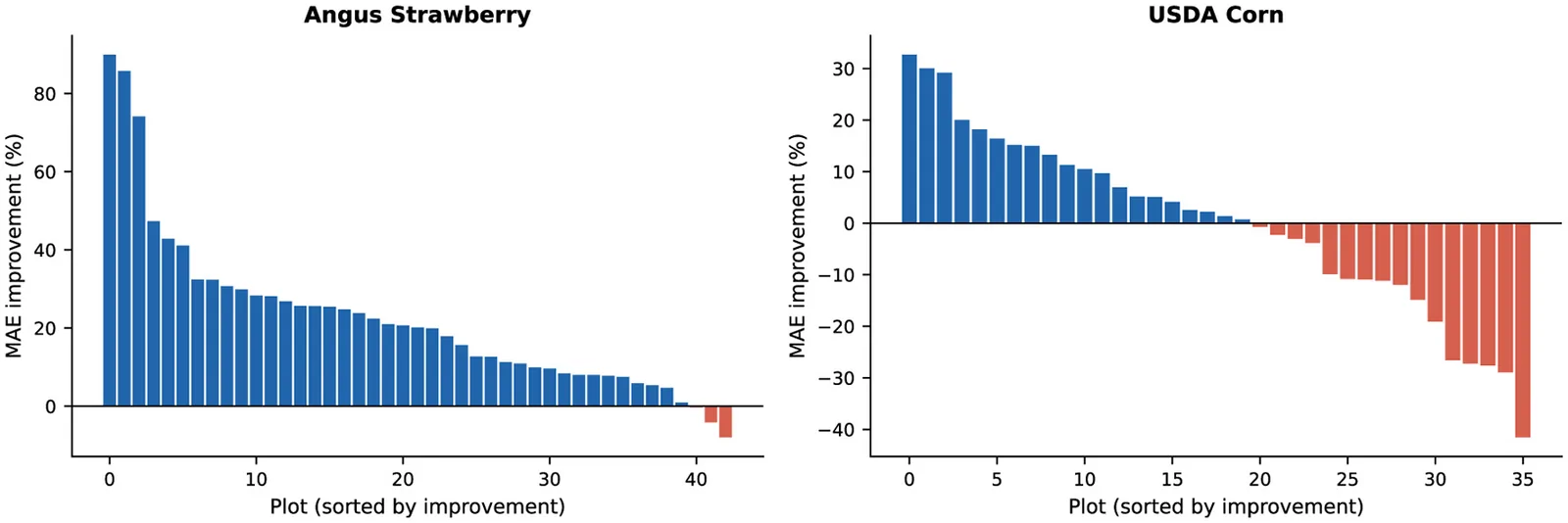
Per-plot MAE improvement (%) for XGBoost + Llama 3.1 8B, sorted descending. Blue bars indicate improvement; red bars indicate degradation.

### 6.1 Generalizability and broader applicability

The agent framework was designed to be dataset-agnostic. The tool set encodes seasonal reasoning through seasonal structure, phase detection, decay estimation, and historical range validation. None of these have been hard coded to match the yields of the dataset used, or strawberries in general. The USDA corn results support this: the same agent with no modification to the tools or prompting, was able to produce consistent improvements on a structurally similar but agriculturally distinct crop, harvested at a different scale, in a different country.

The system requires at least weekly yield records and a seasonal growth curve with an identifiable peak and post-peak decline. This structure is common across soft fruit and most field crops, and the USDA corn dataset confirms the framework transfers across crops sharing this structure. Crops with irregular yield patterns or continuous production without a clear seasonal cycle, such as greenhouse tomatoes or year-round herb production fall outside this assumption. For these crops, the phase detection tool, which assumes a seasonal peak followed by decline, would need to be replaced or supplemented with a structure appropriate to the target crop. The remaining tools, bias learning, range validation, and trajectory evaluation are not crop-specific and would not require modification.

### 6.2 Limitations

#### 6.2.1 Computational overhead

The agent introduces substantial computational cost relative to the base forecasting model, as detailed in Section 5.8. This overhead stems from the nature of the ReAct loop, which issues one or more LLM calls per prediction step. All inference was performed locally on a single GPU via Ollama; wall time is therefore hardware-dependent, and token counts provide a more portable measure of computational demand. In a production setting this cost could be reduced by batching calls or using a faster inference backend, however the agent is not suitable for real-time or low-latency deployment without further optimization.

#### 6.2.2 Dependence on LLM capabilities

The quality of the corrections made by the agent is directly tied to the capabilities of the underlying language model. Smaller models, such as those evaluated in this work, may occasionally produce incorrect tool sequences or fail to appropriately weight the numerical context they are provided with. This means the performance of the system is not solely determined by the reasoning encoded in the toolset, but also by how reliably the model follows structured instructions. This can mean that results are not fully reproducible across different model versions. This is demonstrated by the inferior performance shown when using the LLaVA 13B model as the refinement agent.

#### 6.2.3 Explainability of agent decisions

While each individual tool call can be inspected, the overall correction is computed from the LLM synthesizing multiple tool outputs, which is not fully transparent. A fixed heuristic correction method is auditable and guaranteed to behave consistently, whereas the agent may reach the same conclusion via different reasoning paths on different runs. In a commercial setting, where a grower may want to understand why a forecast has been adjusted, this is a meaningful limitation.

## 7 Conclusion

In this paper, a structured LLM agent framework for *post-hoc* correction of agricultural yield forecasts is presented, designed for the data-constrained conditions that characterize commercial soft fruit production. Rather than addressing the scarcity of farm data by acquiring additional inputs, the agent reasons more effectively over what is already available, applying crop-grounded and statistical logic as a correction layer on top of an existing forecasting model.

The results demonstrate consistent improvements across three base models on the strawberry dataset, with more modest and mixed gains on the USDA corn dataset, where aggregate improvements mask considerable plot-level variation (Figure 6). The largest gains occur where the baseline model diverges most from expected seasonal behavior, confirming that the agent's value lies in correcting systematic errors that the base model lacks the context to fix itself. Llama 3.1 8B produced the strongest LLM-based corrections across all configurations, while the Deterministic baseline was competitive with or outperformed it on XGBoost and Random Forest, suggesting that domain-tool correction alone recovers much of the available gain.

The ablation study identifies phase detection as the load-bearing component of the pipeline. Without it, corrections become unreliable across both datasets. The remaining tools contribute incrementally to the overall correction quality.

In the settings evaluated here, the framework requires no retraining, no additional data collection, and no modification to the base forecasting model. The corn results were produced with no modification to the agent, tools, or prompting. While the current implementation requires approximately 2.8 h per dataset run, this is well-suited to the weekly operational practices of commercial soft fruit production, where corrections can be computed overnight or during off-peak hours and delivered before the working day begins. Within the seasonal agricultural forecasting settings evaluated here, this positions agent-based *post-hoc* correction as a practical complement to existing forecasting pipelines.

## Data Availability

The original contributions presented in the study are included in the article/supplementary material, further inquiries can be directed to the corresponding authors.
